# Supplementary material for: Components of clean delivery kits and newborn mortality in the Zambia Chlorhexidine Application Trial (ZamCAT): An observational study
Source: PLoS Med. 2021 May 5;18(5):e1003610. doi: 10.1371/journal.pmed.1003610 (PMC8133479; doi:10.1371/journal.pmed.1003610)
Supplement: S1 Table — (DOCX) [file pmed.1003610.s002.docx]

# S1 Table. Clean delivery kit (CDK) use by component and perinatal mortality rate

| CDK Component | All locations usage (n=37619) | Home usage (n=13591) | Facility usage (n=24028) | All location user PNMR (deaths/1,000 live births) | All location non-user PNMR (deaths/1,000 live births) |
| --- | --- | --- | --- | --- | --- |
| Soap | 27805 | 11629 | 16176 | 21.9 | 42.1 |
| Gloves | 36411 | 13191 | 23220 | 23.6 | 133.3 |
| Cord clamp | 35138 | 11936 | 23202 | 18.3 | 153.2 |
| Plastic sheet | 36020 | 13335 | 22685 | 23.5 | 109.4 |
| Razor blade | 32859 | 13222 | 19637 | 20.5 | 73.5 |
| Candles | 12936 | 7388 | 5548 | 17.1 | 32.5 |
| Matches | 12722 | 7316 | 5406 | 17.2 | 32.3 |

# *PNMR: Perinatal mortality rate
