# Supplementary material for: Components of clean delivery kits and newborn mortality in the Zambia Chlorhexidine Application Trial (ZamCAT): An observational study
Source: PLoS Med. 2021 May 5;18(5):e1003610. doi: 10.1371/journal.pmed.1003610 (PMC8133479; doi:10.1371/journal.pmed.1003610)
Supplement: S2 Table — (DOCX) [file pmed.1003610.s003.docx]

# S2 Table. Adjusted logistic regression – association between clean delivery kit (CDK) components and perinatal mortality stratified by delivery location

|  | Total | | | Home deliveries | | | Facility deliveries | | |
| --- | --- | --- | --- | --- | --- | --- | --- | --- | --- |
| CDK | OR | p-value | 95% CI | OR | p-value | 95% CI | OR | p-value | 95% CI |
| Soap | 0.91 | 0.25 | 0.78 - 1.07 | 0.79 | 0.14 | 0.58 - 1.08 | 0.98 | 0.85 | 0.82 - 1.18 |
| Gloves | 0.76 | **0.03** | 0.59 - 0.98 | 1.11 | 0.7 | 0.66 - 1.88 | 0.96 | 0.82 | 0.71 - 1.32 |
| Cord clamp | 0.14 | **<0.001** | 0.12 - 0.17 | 0.28 | **<0.001** | 0.21 - 0.37 | 0.05 | **<0.001** | 0.04 - 0.07 |
| Plastic sheet | 0.77 | **0.03** | 0.60 - 0.98 | 0.45 | **0.004** | 0.26 - 0.78 | 1.17 | 0.3 | 0.87 - 1.56 |
| Razor blade | 0.55 | **<0.001** | 0.46 - 0.66 | 0.22 | **<0.001** | 0.15 - 0.34 | 0.95 | 0.68 | 0.76 - 1.19 |
| Candles | 0.56 | 0.12 | 0.27 - 1.16 | 1.03 | 0.96 | 0.36 - 2.91 | 0.42 | 0.07 | 0.17 - 1.09 |
| Matches | 1.09 | 0.82 | 0.52 - 2.28 | 0.58 | 0.31 | 0.21 - 1.65 | 1.78 | 0.23 | 0.69 - 4.57 |
| Mom age |  |  |  |  |  |  |  |  |  |
| Less than 20 | Ref |  |  | Ref |  |  | Ref |  |  |
| 20-29 | 0.64 | **<0.001** | 0.55 - 0.76 | 0.58 | **<0.001** | 0.42 - 0.79 | 0.69 | **<0.001** | 0.56 - 0.84 |
| 30-39 | 1.06 | 0.5 | 0.89 - 1.27 | 0.84 | 0.33 | 0.60 - 1.18 | 1.17 | 0.15 | 0.95 - 1.45 |
| 40 or more | 1.24 | 0.19 | 0.90 - 1.72 | 1 | 0.99 | 0.58 - 1.73 | 1.43 | 0.09 | 0.94 - 2.16 |
| Mom education |  |  |  |  |  |  |  |  |  |
| Didn't finish primary | Ref |  |  | Ref |  |  | Ref |  |  |
| Finished primary but not secondary | 1.1 | 0.18 | 0.96 - 1.27 | 0.81 | 0.17 | 0.60 - 1.09 | 1.13 | 0.15 | 0.96 - 1.34 |
| More than secondary | 0.59 | 0.26 | 0.24 - 1.48 | N/a | N/a | N/a | 0.6 | 0.27 | 0.24 - 1.51 |
| No response | 1.65 | 0.16 | 0.82 - 3.32 | 2.48 | 0.1 | 0.85 - 7.18 | 1.57 | 0.35 | 0.61 - 4.03 |
| Sex of child |  |  |  |  |  |  |  |  |  |
| Female | Ref |  |  | Ref |  |  | Ref |  |  |
| Male | 1.41 | **<0.001** | 1.23 - 1.61 | 1.32 | **0.03** | 1.04 - 1.68 | 1.44 | **<0.001** | 1.22 - 1.69 |
| Newborn birth weight |  |  |  |  |  |  |  |  |  |
| Normal | Ref |  |  | Ref |  |  | Ref |  |  |
| Low birth weight | 0.39 | **<0.001** | 0.26 - 0.58 | 0.99 | 0.98 | 0.42 - 2.36 | 0.32 | **<0.001** | 0.20 - 0.50 |
| Very low birth weight | 0.37 | 0.21 | 0.08 - 1.71 | N/a | N/a | N/a | 0.36 | 0.19 | 0.07 - 1.69 |
| Newborn gestational age |  |  |  |  |  |  |  |  |  |
| Term | Ref |  |  | Ref |  |  | Ref |  |  |
| Preterm | 1.74 | **<0.001** | 1.49 - 2.03 | 2.18 | **<0.001** | 1.66 - 2.87 | 1.53 | **<0.001** | 1.26 - 1.86 |
| Very preterm | 5.49 | **<0.001** | 4.34 - 6.93 | 7.12 | **<0.001** | 4.87 - 10.39 | 4.88 | **<0.001** | 3.59 - 6.64 |
| Extremely preterm | 8.39 | **<0.001** | 6.26 - 11.25 | 7.38 | **<0.001** | 4.29 - 12.70 | 9.39 | **<0.001** | 6.57 - 13.43 |
