# Supplementary material for: Components of clean delivery kits and newborn mortality in the Zambia Chlorhexidine Application Trial (ZamCAT): An observational study
Source: PLoS Med. 2021 May 5;18(5):e1003610. doi: 10.1371/journal.pmed.1003610 (PMC8133479; doi:10.1371/journal.pmed.1003610)
Supplement: S3 Table — (DOCX) [file pmed.1003610.s004.docx]

# S3 Table: Adjusted association between timing of newborn death and the use of clean delivery kit (CDK) components – with covariates

|  | Immediate newborn death (<24 hours) | | | Early newborn death (1-7 days) | | | Late newborn death (7-28 days) | | |
| --- | --- | --- | --- | --- | --- | --- | --- | --- | --- |
| CDK | OR | p-value | 95% CI | OR | p-value | 95% CI | OR | p-value | 95% CI |
| Soap | 0.85 | 0.38 | 0.58 - 1.23 | 0.89 | 0.46 | 0.64 - 1.22 | 1.12 | 0.62 | 0.71 - 1.78 |
| Gloves | 0.47 | **0.03** | 0.24 - 0.91 | 0.26 | **<0.001** | 0.16 - 0.39 | 0.79 | 0.64 | 0.31 - 2.07 |
| Cord clamp | 0.57 | **0.05** | 0.32 - 0.99 | 0.41 | **<0.001** | 0.28 - 0.60 | 1.02 | 0.96 | 0.47 - 2.21 |
| Plastic sheet | 0.81 | 0.52 | 0.43 - 1.54 | 0.37 | **<0.001** | 0.24 - 0.55 | 0.55 | 0.12 | 0.26 - 1.18 |
| Razor blade | 0.85 | 0.49 | 0.54 - 1.35 | 0.59 | **0.01** | 0.40 - 0.85 | 0.74 | 0.30 | 0.43 - 1.30 |
| Candles | 0.35 | 0.30 | 0.05 - 2. 56 | 1.67 | 0.39 | 0.52 - 5.29 | 2.46 | 0.20 | 0.62 - 9.71 |
| Matches | 1.19 | 0.86 | 0.16 - 8.82 | 0.59 | 0.38 | 0.18 - 1.90 | 0.50 | 0.33 | 0.13 - 2.00 |
| Mom age |  |  |  |  |  |  |  |  |  |
| Less than 20 | Ref |  |  | Ref |  |  | Ref |  |  |
| 20-29 | 0.81 | 0.31 | 0.55 - 1.21 | 0.52 | **0.00** | 0.38 - 0.72 | 0.65 | 0.05 | 0.43 - 1.00 |
| 30-39 | 1.14 | 0.56 | 0.73 - 1.78 | 0.78 | 0.16 | 0.55 - 1.10 | 0.72 | 0.20 | 0.43 - 1.19 |
| 40 or more | 0.99 | 0.99 | 0.39 - 2.50 | 1.18 | 0.59 | 0.65 - 2.12 | 1.27 | 0.57 | 0.56 - 2.90 |
| Mom education |  |  |  |  |  |  |  |  |  |
| Didn't finish primary | Ref |  |  | Ref |  |  | Ref |  |  |
| Finished primary but not secondary | 1.23 | 0.23 | 0.88 - 1.73 | 0.99 | 0.97 | 0.75 - 1.32 | 1.18 | 0.40 | 0.81 - 1.72 |
| More than secondary | 1.82 | 0.41 | 0.44 - 7.54 | N/a | N/a | N/a | N/a | N/a | N/a |
| No response | 1.87 | 0.56 | 0.23 - 15.32 | 2.13 | 0.20 | 0.68 - 6.74 | N/a | N/a | N/a |
| Sex of child |  |  |  |  |  |  |  |  |  |
| Female | Ref |  |  | Ref |  |  | Ref |  |  |
| Male | 1.55 | **0.01** | 1.12 - 2.16 | 1.32 | **0.04** | 1.01 - 1.71 | 1.42 | 0.06 | 0.99 - 2.04 |
| Newborn birth weight |  |  |  |  |  |  |  |  |  |
| Normal | Ref |  |  | Ref |  |  | Ref |  |  |
| Low birth weight | 0.07 | **0.01** | 0.01 - 0.53 | 1.21 | 0.43 | 0.76 - 1.93 | 3.34 | **<0.001** | 2.00 - 5.57 |
| Very low birth weight | N/a | N/a | N/a | 1.88 | 0.43 | 0.39 - 9.03 | 10.20 | **<0.001** | 2.99 - 34.75 |
| Newborn gestational age |  |  |  |  |  |  |  |  |  |
| Term | Ref |  |  | Ref |  |  | Ref |  |  |
| Preterm | 2.13 | **<0.001** | 1.43 - 3.17 | 1.50 | **0.01** | 1.10 - 2.05 | 1.78 | **0.01** | 1.19 - 2.68 |
| Very preterm | 12.29 | **<0.001** | 7.83 - 19.28 | 5.18 | **<0.001** | 3.35 - 8.01 | 4.17 | **<0.001** | 2.21 - 7.86 |
| Extremely preterm | 18.90 | **<0.001** | 11.02 - 32.39 | 6.60 | **<0.001** | 3.70 - 11.78 | 3.94 | **0.00** | 1.55 - 10.02 |
