# Supplementary material for: Components of clean delivery kits and newborn mortality in the Zambia Chlorhexidine Application Trial (ZamCAT): An observational study
Source: PLoS Med. 2021 May 5;18(5):e1003610. doi: 10.1371/journal.pmed.1003610 (PMC8133479; doi:10.1371/journal.pmed.1003610)
Supplement: Attachment — Submitted filename: Ver 2.2 Response to Reviewers.docx [file pmed.1003610.s005.docx]

-----------------------------------------------------------

Requests from the editors:

*Please structure your abstract using the PLOS Medicine headings (Background, Methods and Findings, Conclusions -- "Methods and Findings" is a single subsection).

***Response: Requested change was made in the abstract.***

*It might be appropriate to add to the abstract (briefly) the context that although conducted within a randomized trial, the randomization was ignored for the purposes of this analysis, with the participants' data pooled across arms (assuming that is correct) - essentially converting it into something akin to a prospective cohort analysis. That could also be spelt out more clearly upfront in the Methods section, potentially.
***Response: Edit was made in the abstract to reflect this (pg. 2, line 31) and in methods (pg. 5, line 130-132).***

*In the last sentence of the Abstract Methods and Findings section, please include a note about any key limitations of the study's methodology - perhaps one of the most obvious relates to the above point, also brought out by reviewers noting that causality cannot be firmly established due to the potential for confounding (which could be expanded on in the main Discussion, as noted by one reviewer, in terms of the potential source(s) and direction of this).
***Response: Requested edit was made in the abstract (pg.2, line 50-52). Further moderations of the conclusion to reflect the inability to establish causality were implemented throughout the paper as well, as other reviewers have pointed out.***

*Please add the trial registration details to the end of the abstract as per in this (published) example - <https://journals.plos.org/plosmedicine/article?id=10.1371/journal.pmed.1002775>
***Response: Trial registration was added to the end of the abstract (pg. 2, line 53).***

*At this stage, we ask that you include a short, non-technical Author Summary of your research to make findings accessible to a wide audience that includes both scientists and non-scientists. The Author Summary should immediately follow the Abstract in your revised manuscript. This text is subject to editorial change and should be distinct from the scientific abstract. Please see our author guidelines for more information: <https://journals.plos.org/plosmedicine/s/revising-your-manuscript#loc-author-summary>
***Response: Author Summary was added to the manuscript.***

*Regarding the point above, and raised by reviewer(s), currently the Discussion section notes - "In summary, this analysis demonstrated the impact of CDKs on reducing newborn mortality..." - and it might be appropriate to reword this, particularly use of "impact".
***Response: Edit was made to moderate the conclusion (pg. 15, lines 397-398).***

*Please complete the CONSORT checklist  (<https://www.equator-network.org/reporting-guidelines/consort/>) and include a copy of the completed checklist as supporting material with the revised paper. Ideally please ensure that all relevant parts of CONSORT are described in the revised paper, although some elements will only be relevant (and can be found) in the original trial publication.
***Response: CONSORT checklist was completed and was added as supporting material to the submission.***

*Please clarify if the analysis reported here corresponds to one laid out in a prospective protocol or analysis plan. Please state this (either way) early in the Methods section.
***Response: This is a post-hoc secondary analysis of data collected in the Zambia Chlorhexidine Application Trial, a cluster randomized controlled trial. We decided to do this additional analysis for two reasons. First, to understand the uptake and use of clean delivery kits in the study; and second, to assess the associations between kit use and health outcomes of newborns. We had added this information to our methods section. (pg. 7, lines 200-203)***

a) If a prospective analysis plan (from your funding proposal, IRB or other ethics committee submission, study protocol, or other planning document written before analyzing the data) was used in designing the study, please include the relevant prospectively written document with your revised manuscript as a Supporting Information file to be published alongside your study, and cite it in the Methods section. A legend for this file should be included at the end of your manuscript.

b) If no such document exists, please make sure that the Methods section transparently describes when analyses were planned, and when/why any data-driven changes to analyses took place. 

c) In either case, changes in the analysis-- including those made in response to peer review comments-- should be identified as such in the Methods section of the paper, with rationale.
***Response:*** ***In this post hoc secondary analysis, we aimed to understand the uptake and use of clean delivery kits in the study; and second, to assess the associations between kit use and health outcomes of newborns. We have added language to the Methods to clarify the approaches used.***

-----------------------------------------------------------

Comments from the reviewers:

Reviewer #1:

General comments
Clean delivery kits may reduce death from neonatal infections so this study assessed the potential associations of use of components of the clean delivery kits with newborn mortality in a large cluster RCT. 
This study assesses associations so causality should not be inferred. The title implies causality, which should be corrected.

***Response: Thank you for this comment. We reviewed the manuscript to moderate our conclusion and not imply causality.***

Perinatal mortality would be a good measure but stillbirths are not reported.

***Response: Please see our response to the comment below for further discussion on stillbirth data.***

Abstract
The abstract should address that the assessment of the use of the components of the CDK by the mothers and was done late. This should caution the interpretation of the results.

***Response: The abstract Methods section was edited to reflect this (pg. 2, lines 35-36)***

Introduction
The rationale is well-justified.
The final sentence should not imply causality.

***Response: The final sentence was edited to reflect this comment (pg.4-5, lines 120-122).***

Methods
The data on use of the components of the clean delivery kits (CDK) was obtained from the mothers four days after delivery. It is unfortunate these data were not collected at delivery because recall bias can severely affect the results. The mothers may not be able to recall details of the use of the CDK. These data were collected after day 1 mortality and most of the first week mortality were known. This can also lead to major biases.

***Response: This potential bias was added to the limitation section of the discussion (pg. 15, line 369-373). However, we feel this bias would be minimal as only 4 days passed between birth to the postnatal visit where CDK use was confirmed, so recollection should be relatively good still. Further, similar data collection methods and timelines were used in case of a death or stillbirth.***

Non-users of specific CDK component was too low to analyze statistically. This is an important limitation which combined with the recall bias and data collection after most of the first week deaths limits the confidence in the results.

***Response: We have added this point to the limitation section of our study: that this was a post-hoc analysis that did not control the use of CDK and there were very high rates of uptake of CDK (pg. 15, lines 376-380). However, we still feel that the strong differences in NMR between users and non-users of specific components – especially gloves, cord clamps, plastic sheets, and razor blades - of CDKs provide an interesting and compelling result that emphasizes the potential importance of CDKs in improving hygienic childbirths to combat newborn infection.***

Stillbirth data are needed as stillbirths could be due to infection and because the distinction between stillbirth and death in the first minutes is difficult to assess. It is essential to report the combined stillbirth data with the day 1 mortality data as well as the stillbirth/first week mortality data (perinatal death) which is ideal for reporting when assessing intrapartum and birth interventions.

***Response: The relevant analyses in our study were re-performed with perinatal mortality, counting the stillbirths as part of newborn deaths in the first 7 days. We found that the overall perinatal mortality (PNMR) was increased, as would be expected with counting additional deaths, but that there remained reduction in PNMR between users and non-users of CDK components. This reduction was especially seen with gloves, cord clamps, plastic sheets, and razor blades. Adjusted logistic regression saw protective association between gloves, cord clamps, plastic sheets, and razor blades and lower perinatal mortality. When stratified by delivery location, home deliveries saw protective association from cord clamps, plastic sheets, and razor blades and perinatal mortality while facility deliveries had association between cord clamps and lower perinatal mortality.***

***Overall, this analysis showed us that the key messages from our study were similar whether counting stillbirths for perinatal mortality or not: that key components of the CDK including gloves, cord clamps, plastic sheets, and razor blades were associated with reduced newborn mortality, and that this association was mainly seen in the first 7 days of life.***

***While a lot of the results were stable with or without counting the stillbirths, we still believe the procedure would be more accurate without inclusion the stillbirths. The use of CDK would not be expected to reduce the risk of intrapartum fetal demise, as there were no components to help with resuscitation or reduce risk of asphyxia. Thus, including stillbirths as part of the dependent variable may not be an accurate representation of the impact of CDKs. Therefore, we have added the perinatal mortality outcome tables as supplementary materials and added a statement in the Methods (pg.6-7, lines 192-194) and Results (pg. 10, lines 264-267; pg. 12, lines 287-294) about similarities of outcomes.***

Results
Infection in an important cause of late neonatal mortality but individual component of CDK use had not significant association with late newborn deaths. Data on use of components of the CDK were collected on day 4 so it is possible that recall bias had nothing to do with late deaths and thus no association.

***Response: As detailed in the response to the comment below, we believe that infection danger signs may not have reliable correlation with CDK’s potential mechanism of reducing infections. It is true that recall bias would have less effect on late outcomes, and we have added to the limitation portion of our study the potential for recall bias. (pg.14, lines 356-359)***

Furthermore, multivariate logistic regression did not show any significant associations between signs of newborn infection at days 1, 4, 10, or 28 and CDK components or any of the covariates. This raises major concerns about the purported association of components of the CDK and neonatal mortality.

***Response: Thank you for the comment. As mentioned in our manuscript (pg.14, lines 351-360), there was subjectivity and recall bias involved in PSBI (potential signs of bacterial infection) monitoring. In addition, as brought up by reviewers, if newborn death occurred prior to field monitor check for PSBI, the categorization of newborn death timeline (immediate, early, late) may not be accurate. As this was not a major component of the original study design, we did not analyze in granular detail to flesh this out. We have added this limitation to the discussion. (pg. 14, lines 356-359)***

It is not reported whether the deaths in the first week disproportionately caused by infections. Data on verbal autopsy and causes of death were collected but not reported.

***Response: The results from cause of death data were added in text form to the manuscript (pg 13, lines 312-319).***

Discussion
The Discussion should address the results as potential associations rather than imply causality which is implied multiple times in the Discussion. Terms such as impact, effect, and benefit should not be used in the interpretation of the results.

***Response: The discussion section was modified in multiple locations to not imply causality and moderate the conclusion.***

-----------------------------------------------------------

Reviewer #2:

Thanks for the opportunity to review your manuscript. My role is as a statistical reviewer so my comments and queries focus on the study design, data, and analysis.
This study presents secondary analyses from a large cRCT aiming to reduce neonatal mortality in Zambia. These include comparing neonatal mortality rates according to use of specific components of a clean delivery kit, maternal and neonatal characteristics. There are some very strong differences in rates of NMR with some of the kit components.
I have put general queries first, followed by questions specific to a particular part of the manuscript (with a page and line reference).

Some of the language about the association between the CDK components and NMR strays into causation e.g. "showed reduction in risk" (L217), there are few other instances of this. These should be rephrased as although these relationships are strong and seem reasonable there is still the possibility of confounding with an unmeasured variable.
***Response: Thank you for the comment. As other reviewers and editors have commented on this as well, we reviewed the manuscript and edited various parts to moderate our conclusions and not imply causality.***

I would also try not to use 'significant' or 'statistically significant', rather focus on the effect estimates and use the p-values to as a demonstration of evidence. The effect sizes are very impressive so it would be a shame not to highlight these.
 ***Response: We went through the paper and tried to remove the above words when possible. We kept it at times for sake of brevity in times where highlighting specific p-values or effect estimates would have been unnecessarily verbose.***

P6. L164. To confirm, the main study used GEEs as there was cluster randomisation, the randomisation status wasn't used in any of these analyses so a standard logistic regression model was used throughout?
***Response: That is correct, the main trial used GEE to adjust for cluster randomization, but this post-hoc analysis used standard logistic regression models.***

P6, L167. 'Confounding by indication' has become a fairly vague term with different interpretations in different sub-fields of health and medicine. It would be more useful to be specific about how you think the confounding would affect your analysis here (keeping in mind PLOS Med has a general audience). 
***Response: The sentence was edited to more clearly explain the reason for initially excluding stillbirths from the analysis (pg. 7, line 208-210).***

P6, L174. Theoretically there are 2**7 = 128 permutations of use of the 7 different components of the CDK. How were the 'key permutations' decided? It isn't clear what the 'analysis of interactions' was - is this the interaction with the other patient and cluster level characteristics?
***Response: Thank you for this comment. While analysis of the interaction for each of the 128 permutations was originally planned, this was impractical due to the small sample size of less frequently used permutations and difficulty of interpretation. Instead, we descriptively looked at the most commonly used combinations, their frequency, as presented in table 2b. The methods were edited to explain this descriptive process (pg.7, lines 214-216).***

P6, L175. Were the risk factors for newborn sepsis that were included as adjustments used in the same form as presented in table 1?
***Response: The covariates for adjustments were the ones listed on pg.7, lines 217-219. Table 1 had maternal marital status as an extra variable for descriptive purposes and delivery locations, which was analyzed in the home vs facility deliveries but not as one of the adjustments.***

P6, L180. Was an analysis plan developed for this secondary analysis?
***Response: Please see our response to the comment from the editors about the post-hoc analysis.***

P6, L189. The missing data strategy (complete-case analysis) should be detailed in the methods. It would also be helpful to see basic summary information that compares participants included vs. excluded on key characteristics (e.g. what is presented in Table 1). I don't think that a complete-case excluding ~2.5% of the original participants is likely to be a problem, but it would be reassuring to see there were no strong differences.
***Response: Description of missing data strategy was added to the methods (pg.7, lines 206-208). The excluded participants’ descriptive characteristics were added to Table 1, including a brief description in the results (pg.8, lines 237-239).***

P9, L238. Was the interaction between delivery location and CDK etc. formally tested with an interaction?

***Response***: ***As described in the response to the above comment, the use of “interactions” was from an older analysis plan that was replaced with a simpler and most descriptive table.***
       
-----------------------------------------------------------

Reviewer #3:

While this study is a secondary analysis that employs data from the primary Zambia chlorhexidine Application Trial, the findings are nonetheless compelling.

Almost 37,000 newborns were included in the analyses which reported on independent rates of the "6 cleans" based upon the provision of clean delivery kits. Independent rates of reduction in mortality for each component contained in the kit have been reported, along with its association on immediate newborn death, early as well as late neonatal mortality.
 
It appears evident from data provided in this paper, utilizing logistic regression analyses, that employment of clean birthing practices has the potential to produce significant reductions in mortality. The impact appears to be meaningful immediately after birth and within 7 days of delivery with no additional reduction in mortality after that time.

The authors appropriately point out the weaknesses inherent in using self-reported data. It would be interesting to determine why cord clamping was not practiced and home births relative to its use at facilities. Suggestions for education and training of community health providers may be important in addressing this difference in future interventions.
***Response: Thank you for this query. We agree it would be interesting to understand why some items (i.e. cord clamps) were used more or less than others based on location of birth. Unfortunately, we do not have the qualitative data to answer this question but we have included in the discussion (pg. 15, lines 393-396).***

The paper would be enhanced by including costs associated with providing the intervention.
***Response: Thank you for your query; we have noted in the (pg. 6, line 166 of the Methods, the cost of the clean delivery kit. However, since the CDK was delivered as part of a larger clinical trial with extensive follow up (one antenatal visit and four postnatal visits) we do not have cost available of delivering the CDK itself.***

Overall, however, there is sufficiently strong evidence presented to expand the use of clean delivery kits as an important component for reducing immediate and early neonatal mortality in Zambia and likely in other sub-Saharan communities.

***Response: Thank you for the encouragement. We do believe this data provides support to clean delivery kit use and provision. We have included this in our conclusions to think about scale up (pg. 16, lines 402-403).***

-----------------------------------------------------------
