# Supplementary material for: Components of clean delivery kits and newborn mortality in the Zambia Chlorhexidine Application Trial (ZamCAT): An observational study
Source: PLoS Med. 2021 May 5;18(5):e1003610. doi: 10.1371/journal.pmed.1003610 (PMC8133479; doi:10.1371/journal.pmed.1003610)
Supplement: Attachment — Submitted filename: Ver 3.2 Response to reviewers.docx [file pmed.1003610.s006.docx]

Requests from Editors:

Please finalize the arrangements for data deposition in a publicly accessible repository.
***Response****:* *De-identified data for this analysis will be available through OpenBU, Boston University’s data repository (*[*https://open.bu.edu/*](https://open.bu.edu/)*). The OpenBU repository has data access policies and procedures consistent with NIH data sharing policies.*

So as to comply with journal style, please adapt the title to "Components of clean delivery kits and newborn mortality in the Zambia Chlorhexidine Application Trial (ZamCAT): an observational study" or similar.
***Response****: Title was updated to: Components of clean delivery kits and newborn mortality in the Zambia Chlorhexidine Application Trial (ZamCAT): an observational study.*

Please quote the study dates in the abstract.
***Response****: Dates were added to abstract (pg. 2, line 33).*

At lines 44-49, please adapt "reduced newborn mortality" to "lower risk of newborn mortality", for example.
***Response****: Requested change was made to abstract (pg. 2, lines 43-48).*

Please add a new final sentence to the "Methods and findings" subsection of your abstract, which should begin "Study limitations include ..." or similar and quote 2-3 of the study's main limitations.
***Response****: Limitations were added to the abstract (pg. 2, lines 48-50).*

Please use the active voice for one or two points in your Author summary (e.g., "We analysed data ...").
***Response****: Syntax was changed to be in active voice (pg. 3, lines 66-73).*

Please state explicitly in your Methods section that the analysis did not have a protocol or prespecified analysis plan (assuming this is the case); are you able to state that there were no data-driven changes in the analysis plan?
***Response****: Requested line was added to methods section (pg. 5, line 134).*

Please avoid language implying causality throughout the text. For example, at line 400 rather than "were associated with the greatest reduction ..." please adapt the text to "were associated with the lowest risk of ..." or similar.
***Response****: We reviewed the manuscript and edited language implying causality to the best of our ability.*

Please use the following style throughout the text, except where a number is used to start a sentence: "All 7 components ...".
***Response****: We reviewed the manuscript and requested edits from text to numerals were made throughout the text.*

Please remove the information on study funding from the Acknowledgements section at the end of the main text. This information should appear only in the article metadata, via entries in the submission form.
***Response****: Funding information was removed as per request. The language regarding support from Fogarty International Center was retained per the fellowship’s request, as they did not fund the initial trial and provided non-financial support as well.*

Please adapt the attached CONSORT checklist so that individual items are referred to by section (e.g., "Methods") and paragraph number rather than by page or line numbers, as the latter generally change in the event of publication.
***Response****: CONSORT checklist was edited to be organized by paragraph number in relation to each section as requested.*

Please rename the file "S1_CONSORT_Checklist" or similar and refer to it in your Methods section by this label.
***Response****: Requested file name change was made.*

Comments from Reviewers:

*** Reviewer #2:

Thanks for the revised manuscript and replies to my original queries. This is an interesting and useful manuscript and overall I recommend it. There are a few minor changes I recommend.

The language around causality is improved.  In L401 I think 'connection' is still too strong, 'association' would be more accurate.

***Response****: Thank you for the comment. As commented above, we reviewed the manuscript and changed wording to not imply causality, to the best of our abilities.*

For the most common combinations, I would add change the table description from 'top' to 'most frequent' as this is more specific and not every reader will be examining the methods as closely as I like to.

***Response****: Requested edit was made to table description (pg. 10).*

 Women excluded from the analysis for missing data are broadly the same as those included - there are more older women, and more likely to deliver in a hospital, but I don't see this as being problematic with 1583 missing from the original sample.
***Response****: Thank you for the comment, we agree with this assessment.*

L297. 'Multivariable' is a more suitable description with one outcome and several covariates.
***Response****: Requested edit was made (pg. 12, line 301).*

L308. Is the adjusted analysis shown somewhere? If in an appendix (I couldn't find it) please refer to this.  This should be included somewhere, either by expanding Tab 4 or including in an appendix.
***Response****: We did not include the full covariate relationships on the main manuscript for brevity’s sake, as there were no major takeaways. We added the full adjusted table as another one of the supplemental tables for reference.*

Supplemental Table 1 didn't appear in the document when I downloaded it.

***Response****: From our end it was uploaded, but supplemental material may not have been automatically downloaded as part of default manuscript submission.*
